# Why and how science students in the United States think their peers cheat more frequently online: perspectives during the COVID-19 pandemic

**DOI:** 10.1007/s40979-021-00089-3

**Published:** 2021-11-17

**Authors:** Lisa L. Walsh, Deborah A. Lichti, Christina M. Zambrano-Varghese, Ashish D. Borgaonkar, Jaskirat S. Sodhi, Swapnil Moon, Emma R. Wester, Kristine L. Callis-Duehl

**Affiliations:** 1 Donald Danforth Plant Science Center, St. Louis, MO USA; 2 Interdisciplinary Science Learning Laboratories, University of Delaware, Newark, DE USA; 3 Department of Psychology, Rutgers University-Newark, Newark, NJ USA; 4 School of Applied Engineering & Technology, New Jersey Institute of Technology, Newark, NJ USA; 5 Mechanical & Industrial Engineering, New Jersey Institute of Technology, Newark, NJ USA; 6 Department of Biology, St. Louis University, St. Louis, MO USA

**Keywords:** Academic dishonesty, Assessment, Cheating, COVID-19, Emergency, Integrity, Planned behavior theory, United States

## Abstract

Academic integrity establishes a code of ethics that transfers over into the job force and is a critical characteristic in scientists in the twenty-first century. A student’s perception of cheating is influenced by both internal and external factors that develop and change through time. For students, the COVID-19 pandemic shrank their academic and social environments onto a computer screen. We surveyed science students in the United States at the end of their first COVID-interrupted semester to understand how and why they believed their peers were cheating more online during a pandemic. Almost 81% of students indicated that they believed cheating occurred more frequently online than in-person. When explaining why they believed this, students touched on proctoring, cheating influences, and extenuating circumstances due to COVID-19. When describing how they believed cheating occurred more frequently online, students touched on methods for cheating and surreptitious behavior. The student reasonings were associated with four theories (game theory, Kohlberg’s theory of moral development, neutralization theory, and planned behavior theory) that have been used to examine academic dishonesty. Our results can aid institutions in efforts to quell student concerns about their peers cheating during emergencies. Interestingly, most student beliefs were mapped to planned behavior theory while only a few students were mapped to neutralization theory, suggesting it was a novel modality of assessment rather than a pandemic that shaped student perceptions.

**Supplementary Information:**

The online version contains supplementary material available at 10.1007/s40979-021-00089-3.

## Introduction

In March 2020, most United States higher education institutions were forced to move all their courses to online instruction within a matter of days and continue remotely for the rest of the semester due to the COVID-19 pandemic (Crawford et al. 2020). We refer to this semester that was partially in-person and partially remote as the first COVID-interrupted semester. As undergraduate students in the United States were asked to leave their campus housing and adapt to remote learning, they experienced an increase in depression and anxiety symptoms (Huckins et al. 2020) which can drive students to make poor decisions and disengage from their coursework (Mazer et al. 2014; England et al. 2017).

### STEM academia during COVID-19

For many science professors and students, March 2020 was their first exposure to online learning assessments, and concerns of cheating immediately centered on high-stakes online exams. While learning assessments take a variety of forms in undergraduate science courses including research papers, group presentations, and low-stakes quizzes, high-stakes exams are known to cause test anxiety that disproportionately affects the achievements of underrepresented populations, such as women, in science courses (Eddy et al. 2014; Harris et al. 2019). As classes moved online, some U.S. institutions cancelled midterm exams to provide instructors with more time to adjust their classes, thereby putting more pressure on students to perform well on their final exams (Dietrich et al. 2020), thus increasing the already high stakes final to weigh even more on the students’ final grade and increasing student stress and potential pressure to cheat. Finally, science students were also concerned about having to learn to use the online testing software and encountering technological glitches that may occur during their online exams (Dicks et al. 2020; Holton 2020; Petillion and McNeil 2020), also increasing the students’ stress levels around taking and performing well on exams in new environments with unfamiliar systems.

In addition to becoming more anxious and depressed early in the pandemic, STEM students also became less engaged in participating in their courses (Huckins et al. 2020; Perets et al. 2020; Wester et al. 2021). One study that evaluated the engagement of students in science courses during the first COVID-interrupted semester found a decline in their value in science (Wester et al. 2021), an early outcome of the pandemic that runs counter to efforts to improve retention rates in STEM. Long-standing inequities among STEM undergraduate students in the United States (e.g., financial, social), were made conspicuous during remote learning by discrepancies between access to internet and equipment and the ability to secure safe and quiet study space (Barton 2020; Castelli and Sarvary 2021). Even algorithms used by proctoring software were biased against students who behaved outside of the programmed “norm” during test taking, incorrectly flagging autistic and blind students for their movements and parents for the noise of children (Swauger 2020).

### COVID-interrupted education and cheating

For many STEM faculty members, the semester of March 2020 was their first time teaching online, and those teaching laboratory or field-based courses faced the additional challenge of rapidly creating remote alternatives to the hands-on learning experiences traditionally embedded in their courses (Barton 2020; Holme 2020; Walsh et al. 2021). Because instructors had to rapidly transition their classes online, many saved time by maintaining the assessments originally designed for an in-person curriculum (Dietrich et al. 2020; Eaton 2020; Rupnow et al. 2020). As many instructors and students encountered online learning for the first time during the COVID-19 pandemic, discussions around academic integrity increased in frequency, most noticeably cheating in online tests (e.g., Supiano 2020). With test score averages increasing after the transition online, faculty members began to suspect that students were cheating in their online exams (Eaton 2020). During the same period of COVID-interrupted education, the use of commercial file websites (e.g., Chegg) by students increased by almost 200% between 2019 and 2020, with chemistry experiencing an especially high increase (Lancaster and Cotarlan 2021), supporting faculty’s perception of increased cheating among students during the pandemic.

Efforts to mitigate cheating on high stakes online exams during the first COVID-interrupted semester varied. Some universities purchased test proctoring software which blocked web browsing and used student webcams to monitor for “suspicious” behavior (Holton 2020; Swauger 2020). Cheaper and more pedagogically robust alternatives to curb cheating during the first COVID-interrupted semester included open-book exams, group oral exams, and higher-order thinking questions (Dingwall 2020; Goodman 2020; Nguyen et al. 2020), and a survey of Australian students confirmed that they perceived these assessments as more difficult to cheat (Reedy et al. 2021).

### Student perceptions

Given the personal and academic stress that students were encountering in the early months of the COVID-19 pandemic (Huckins et al. 2020), along with the prevalent conversations surrounding cheating online and the continued pressure to perform well on tests, the perception that their peers were cheating during the first COVID-interrupted semester could further defray the mental health and behavioral engagement of science students (van Zyl and Thomas 2015; Fontaine et al. 2020; Putarek and Pavlin-Bernardic 2020). In order to reduce science student concerns over their peers cheating during emergency disruptions to education, we must identify why students perceive their peers as more likely to cheat in the remote learning environment.

In the early months of the pandemic, university students from across disciplines grew more concerned that their peers were cheating after their classes moved online (Daniels et al. 2021), but the researchers did not evaluate why student concerns increased. Improving our understanding of why students grew more concerned about their peers cheating after their classes transferred online due to a pandemic is important because a student’s perceptions can influence their behavior (Ajzen 2002; Pajares 1992; Spaulding 2009). Research indicates that certain external factors like curved grading and the idea that “everyone else is doing it” tend to encourage cheating behaviors. Additionally, survey data demonstrate that students are less likely to cheat with good instructor interaction and a socio-cultural belief that their peers are primarily honest (Richardson and North 2013; Carpenter et al. 2005; Turner and Uludag 2013). Being aware of other students’ cheating behaviors, particularly in online classes through group chats or other methods, may lead students to cheat who would not have otherwise cheated because of the added pressure and perceived lack of repercussions online (Finn and Frone 2004; van Zyl and Thomas 2015).

### Research questions

By identifying why and how science students believed their peers cheat more online during the COVID-19 pandemic, we can improve our recommendations to science departments for effective interventions that minimize student concerns over academic integrity both during and outside of emergency events. As the first COVID-19-interrupted semester came to a close in May and June 2020, we surveyed biology and chemistry students to

1. Evaluate if science students believed the pressure or willingness to cheat varied between education modalities (i.e., online vs. in-person).
2. Confirm most science students thought their peers were cheating more online.
3. Identify themes that emerge when students explain why and how they believe their peers are cheating more online.
4. Map themes to academic dishonesty theories to place the perceived behaviors within the context of cheating theory.

## Methods

### Surveys and quantitative analysis

Biology faculty were solicited in March 2020 using biology (e.g., Ecological Society of America) and STEM education research (e.g., Society for the Advancement of Biology Education Research) listservs (email list that distributes to all scientific society members) to distribute our end-of-semester Qualtrics survey to their students. Faculty distributed a link to the Qualtrics survey at the end of their term (Internal Review Board Approval Number_2020_02). The students signed a consent form at the beginning of the survey, all questions were voluntary, and the participants could opt-out at any time during the survey. Due to the nature of survey solicitation via listserv, we do not know how many students were sent the survey link by their instructors, and therefore cannot calculate the response rate. A total of 299 students completed the survey from 31 different institutions across the United States, including research-intensive universities, Master’s and PhD granting institutions, primarily undergraduate institutions, and community colleges (see Supplementary Table A for details). Survey respondents included 90 men and 202 women, with approximately 50% of students attending research-intensive, R1 institutions. Additional demographic details can be found in Supplementary Table B.

To evaluate how student perceptions of cheating varied between in-person and emergency online modalities, students were asked four three-point Likert scale questions, one multiple-choice question, and one open-ended question. Because these questions were part of a larger survey on the impacts of COVID-19, we chose brevity over a longer validated survey. For each modality, two Likert scale questions grouped together in a matrix format asked students to use their experiences talking to friends and classmates to rate the frequency of 1) willingness to cheat and 2) pressure to cheat (Table 1), which serve as cheating perception constructs in our study.

**Table 1:** The four Likert scale cheating questions posed to students at the end of their first COVID-19 semester. The percentage of students (*n* = 299) who selected each of the three options is also included

*Survey Question: Within your experience of talking to friends and classmates, what is your experience with these activities in online classes:*			
	Rarely	Sometimes	Often
Willingness to cheat	54.18%	31.44%	14.38%
Pressure to cheat	60.53%	27.43%	12.04%
*Survey Question: Within your experience of talking to friends and classmates, what is your experience with these activities in face-to-face classes:*			
	Rarely	Sometimes	Often
Willingness to cheat	83.56%	13.42%	3.02%
Pressure to cheat	82.10%	15.20%	2.70

For statistical analyses, Likert responses were converted to numbers so that rarely = 0 and often = 2. For each of the cheating perception constructs, we calculated the change (online - face-to-face) and used the sign test from the “BSDA” R (version 3.6.0) package to evaluate if there was significant change between modalities (Roberson et al. 1995; Arnholt and Evans 2017). The multiple-choice question asked students if they thought cheating happened more or less frequently in online classes than face-to-face classes. To confirm that most science students believed cheating was occurring more often online during the first COVID-interrupted semester, the number of students who responded “more” and “less” were evaluated for significant variance (significant variance accepted at *p*-values < 0.05) from equal distributions using the Chi-squared test for given probabilities in R (v 3.6.0). Finally, an open-ended question asked “If [cheating happens] more frequently in online classes than face-to-face classes, why? How?”

### Qualitative coding

Qualitative coding was completed for 197 student responses to the open-ended question. We removed any open-ended responses that only included neutral responses (*n* = 3) or responses that were given by students that indicated they believed that cheating occurred less frequently online (*n* = 3). The open-ended responses were coded by two separate coders. The coders initially used an inductive analytic approach. After the 14 codes were determined, the coders independently coded all the open-ended responses. Each response could be coded for multiple codes based on students explaining in multiple ways *why* and *how* they thought students cheated more frequently online. Once the two coders completed their coding, they compared codes and discussed any discrepancies, and agreement was reached for each response using the applied comparative coding methods.

**Fig. 1:**
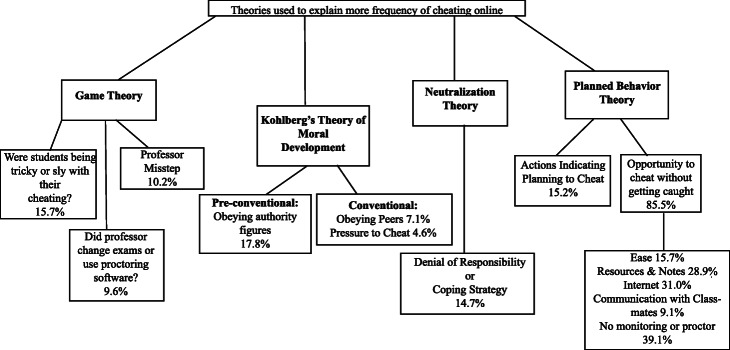
The four academic dishonesty theories connected to the coding of open-ended responses for why and how cheating occurs more online than in face-to-face courses in the first COVID-interrupted semester

As the coding scheme was developed, we evaluated a recent review of the more than 20 academic dishonesty theories (Madara et al. 2016) to identify which theories aligned with the codes that were emerging from our student responses. We identified four cheating theories that aligned with the coding themes: 1) game theory, 2) Kohlberg’s theory of moral development, 3) neutralization theory, and 4) planned behavior theory. We mapped our themes to these four theories in order to place the students’ perceptions of their peers’ behavior within the context of cheating theory (Fig. 1).

In the context of *game theory*, the student and the professor serve as the players that strategize against one another in an effort to maximize payoff (DiPietro 2010). The professor will take steps to reduce cheating, which then causes the student to change their cheating tactics, which may in turn cause the professor to further adjust assessment strategies to reduce cheating. We placed our codes that described surreptitious behavior, or how professors handled the move to courses online under game theory. The statements incorporating the behaviors of the professors were divided into two categories: 1) professors changed methods when moving online or 2) professors made a misstep by not using “appropriate” methods to reduce cheating (Fig. 1).

*Kohlberg’s theory of moral development* lays out stages of development in which the morality of an individual is influenced by various parties. In the preconventional stage, morality is established by authority figures including parents and teachers. In the conventional stage, morality is defined by actions that maintain or improve personal and societal relationships (Levine et al. 1985). In Kohlberg’s theory of moral development, the codes were placed under preconventional and conventional stages. In the preconventional stage, we placed our responses that coded to the presence of an authority figure (example: professor, instructor, or teaching assistant) in the classroom. Under the conventional stage, we placed responses that were coded for statements about the pressure to cheat or the presence of peers in the same room when taking exams in-person (Fig. 1).

*Neutralization theory* posits that individuals rationalize behavior that they know is traditionally immoral (Sykes and Matza 1957). Four neutralization techniques for cheating were identified in undergraduate students: deny responsibility (i.e., didn’t mean to cheat; circumstances beyond their control), deny consequences, blame the professor or institution, or refer to an alternative value system (i.e., helping a friend; Storch et al. 2002). The responses that were coded for outside sources, especially when language appeared about denial of responsibility or discussion of coping strategies by cheating, were placed under the neutralization theory (Fig. 1).

The *planned behavior theory* posits that cheating happens when a student has both the intention to cheat and the opportunity (Ajzen 1991). Intention is composed of a positive attitude toward the behavior, perception of cheating, and perceived easiness of cheating (Ajzen 2002). We placed responses that coded for action indicating plans to cheat or opportunities to cheat under the planned behavior theory (Fig. 1). The action indicating a plan to cheat was based on the language of desire and temptation. The opportunities to cheat were mostly coded for examples on how they cheated (e.g., using resources, internet or communicating with classmates) with one reason (e.g., not being monitored) for why. We included quotes from the students to represent the different coding and themes they relate to below.

We calculated the number of responses for each theory, and then analyzed the responses to determine the number of different theories that were in each response. For responses with more than one theory, we then determined if specific theories seemed to be more likely to pair together. The multiple theories coded to individual responses demonstrate that students did not only have one perception of cheating but could describe in short responses many reasons for why and how others might cheat during online exams.

## Results

Each cheating perception construct changed between modalities so that willingness and pressure to cheat were both perceived to occur more often online (all sign test *p* < 0.001). The more extreme change was willingness to cheat, with 83.56% of students rating it “rarely” in face-to-face classes but only 54.18% rating it “rarely” online (Table 1). When asked to compare cheating between the two modalities, 80.9% of students selected that cheating occurred more frequently online than in face-to-face classes (Table 2). The distribution of students who responded more frequently was significantly higher than random (Chi-squared *p* < 0.001). Responses to the overarching open-ended question had three themes for why students believed their peers cheat more online, and two themes for how students believe their peers cheat more online.

**Table 2:** Student survey responses regarding cheating perceptions (online versus face-to-face)

	Total	Percent
**More Frequently Online**	242	80.94
**Less Frequently Online**	48	16.05
**N/A**	9	3.01

### Why students believe cheating is more frequent online

When students explained *why* they believed cheating occurred more frequently online, three broad themes emerged including proctoring, cheating influences, and extenuating circumstances and/or outside sources.

#### Proctoring

One of the main themes resulting from the open-ended questions was proctoring/lack of proctoring. Not having a proctor or being monitored during exams came up in 39.1% of the students’ responses (Fig. 1, Supplementary Table C). One student explained

“There is no way to monitor what is in the students' environment throughout the entire test” (No Proctoring, Planned Behavior Theory).

Students (17.8%) posited that not being in a classroom for professors, instructors or teaching assistants (TAs) to monitor student behavior during exams increases student cheating (Fig. 1, Supplementary Table C). A few students (4.6%) felt that not being in a room with their peers could lead to more cheating online (Fig. 1, Supplementary Table C). One student wrote about the potential influence of not being physically in the classroom and stated

“When taking online tests or completing projects you can have the notes beside of you [sic]. In class this is not possible when the teacher is watching you take the tests, or complete the assignments. You can ask other students for help on individual assignments when in the online format because they can complete the assignment before you do. Then they have seen the assignment and know what is asked/expected” (Professor Not Physically Present, Kohlberg’s Theory of Moral Development).

A student discussed not being around peers and wrote

“You are surrounded by more people in class” (Pressure to Not Cheat with Peers in Room, Kohlberg’s Theory of Moral Development).

Students (10.2%) wrote about professors not using the proctoring technology once the courses moved online, which could result in more cheating (Fig. 1, Supplementary Table C). For example, a student stated

“A lot of professors just don’t use the 3rd party apps for test/quiz monitoring and students don’t feel like classes are worth as much effort” (Professor Misstep, Game Theory).

#### Cheating influences

Another theme that arose from the students’ responses was different influences (e.g., pressure, desire, temptation or ease) to cheat since moving to online. Students (7.1%) stated that pressure to cheat was higher since moving online, especially because there was a lack of motivation (Fig. 1, Supplementary Table C). Students’ responses included terms like ‘temptation’ and ‘desire to cheat’ as reasons for online cheating. The overall ease of cheating was included in reasons for why cheating online occurred more than in face-to-face courses in 15.7% of the responses. A student included this statement

“I think there is more cheating in an online system because nobody is there to monitor you and you are have [sic] easy access to notes and the internet. Also, there is more motivation or pressure to cheat due to lack of motivation for class work, but still desiring a good grade” (Pressure to Cheat, Kohlberg’s Theory of Moral Development).

In one student response that touched on their peers’ intention to cheat, they gave an example of temptation:

“I think there greater [sic] temptation to cheat at home because there isn’t the pressure that professors add by being there. I do, however, think that students who cheat at home are equally capable of cheating in class” (Actions Indicating Plans to Cheat, Planned Behavior).

For an example of perceived ease in cheating, a student stated

“It's easier to cheat, and people have pressures that make it harder to concentrate on work. That being said, I haven't encountered cheating, so what I said there is basically just a hypothesis” (Ease of Cheating, Planned Behavior Theory).

#### Extenuating circumstances

A theme that occurred throughout the responses was extenuating circumstances from the pandemic and outside sources, especially the way professors handled the transition online. Students referenced the impact that outside stressors and being sent home to finish their studies for the COVID-interrupted semester might have had in decreasing student motivation and/or student well-being. For example, a student stated

“It is not happening more frequently just because classes are online which makes it "easier to cheat", but students are cheating for several reasons more so now than before. In classes where the same or more amount of work is expected (because we "have more time now") is when students cheat to get by. Students are becoming overwhelmed, losing motivation to keep up, and even despairing when there are several other stressors going on in their lives. Feeling the need for good grades in order to be good enough has always been a pressure to cheat. But now there is no motivation to keep up or pay attention because we are stuck at home waiting for the next day to be the same. There are no small daily positives of regular school life (like seeing your friends or talking with people or even just being physical activity by walking to class) and no external motivation to finish assignments (ie. "Once I finish this project, then I can go out with my friends" etc.). The classes that have the most cheating are the ones that hold standards for grades and amount of assignments higher than their students' well-being” (Neutralization Theory).

Students also included how the professor handled the transition as outside sources that could result in more cheating. Students felt that professors did not change the course or were not clear on expectations. The students also included statements about being online and that online is harder or not as clear as being in face-to-face courses. One student mentioned

“It is complicated because the word "cheating" is ambiguous here. Some professors are allowing open-note quizzes/tests or just didn't say we couldn't use them. Is that "cheating" if we do? I take a lot of online classes already and usually there are weekly quizzes. I have used my notes to take those quizzes (and tests) if the professor does not specify that we can't use them. Never had an issue” (Neutralization Theory).

Another student stated

“I feel as though that online classes are considered less 'serious' by students in my grade or any active year of undergraduate studies. The classes are often structured poorly and things typically aren't as smooth” (Neutralization Theory).

Students (9.6%) also wrote about how professors changed their courses to make it circumvent cheating by setting time limits and having open note and book exams (Fig. 1, Supplementary Table C). One student wrote

“It's just easier. It's almost impossible for professors to prevent. It's best when professors have open-book open-note exams because then everyone starts on a more level playing field. At the bare minimum, it would be very hard not for a student to check their notes during the exam if there was nothing to stop them. Assuming that exams are open-book open-note (as they should be), students with a desire to cheat can easily reach out to other students taking the same exam to exchange questions and answers on their phones, even if a lockdown browser is being used. I think that the most optimal solution would be to have exams timed so that they can only be taken at one time during the day, and for teachers to use an online proctoring service like ProctorU, but many don't even bother, or don't have the technical resources.”

### How students believe cheating occurs more online

When students explained *how* they imagined cheating occurred more frequently online, two main themes emerged: putative methods for cheating and surreptitious behavior. When describing putative methods for cheating online, three major methods were discussed: the internet, accessible resources, and communicating with others. Students (31.0%) discussed the possibility that their peers were using the internet and looking up answers online during the exam (Fig. 1, Supplementary Table C). For example, one student stated

“The internet is at our finger tips and the temptation is high” (Internet, Planned Behavior Theory).

Similarly, 28.9% of students cited their peers’ ability to easily use resources, textbooks and notes during an online exam (Fig. 1, Supplementary Table C). One student wrote that students were

“More likely to check the book to verify answers on quizzes or exams” (Resources and Notes, Planned Behavior Theory).

The potential that their peers could communicate with classmates during exams came up in 9.1% of the students’ responses (Fig. 1, Supplementary Table C). While they often referred to phones, some respondents also cited Zoom as a means of communication during an exam. A student stated

“Some classes do not require a webcam, where students work on the test together when it's not allowed” (Communicating with Classmates, Planned Behavior Theory).

Some student responses (28.9%) discussed more than one putative method for cheating online and even discussed unlimited methods to cheat during online courses. For example, a student stated

“Because in a remote setting, it is easier to have other tabs, notes, books, flashcards, material open to help aid in the quizzes, tests, or discussions. Additionally, you can call your friends and brainstorm together what the answer might be. There are unlimited avenues to allow cheating in online formatting. Face to face does not allow any of that to occur with my professor and class structure.”

Students in 15.7% of the responses wrote about potential surreptitious behavior of their peers (Fig. 1, Supplementary Table C). Students wrote about circumventing proctoring technology by having multiple screens, being tricky, and hiding notes. A student wrote

“I know some people who smear their webcam to decrease the quality of proctored exams so their eyes can move around to cheat more easily; hiding cheat sheets not visible by webcam; collaborating with others”

On the potential utility of multiple screens, a student wrote

“so many screens and devices, it's easy to circumvent anti-cheating methods. Though some might just call that resourceful learning.”

Students also discussed how lockdown browsers have limitations. A student stated

“Even with proctoring systems such as lockdown browsers, I have heard of methods students use to cheat such as having notes on their lap or not using a completely blank sheet of scratch paper.”

### Themes to theories

**Fig. 2:**
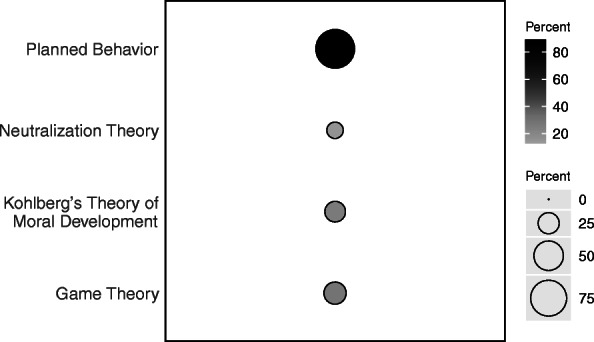
Percent of students using the four academic dishonesty theories when explaining why they believe cheating occurs more frequently online

The majority of students’ open-ended responses (87.3%) included themes that were mapped to *planned behavior theory* (Fig. 2). Planned behavior theory includes both opportunity to cheat and intent to cheat, and students were more than five times as likely to discuss the opportunity their peers had to cheat than intention (85.8% vs. 15.2%; Supplementary Table C). When explaining their peers’ opportunities to cheat, five themes emerged, in order of frequency: no proctor, access to internet, access to resources and notes, easier to cheat online, and communication with classmates (Fig. 1, Supplementary Table C).

More than a quarter of students’ open-ended responses (27.9%) included themes that were mapped to *game theory* (Fig. 2), including ways their peers could work around the system and how professors did or did not deal with possible cheating (Fig. 1). Almost a quarter of students’ open-ended responses (23.9%) included themes that were mapped to *Kohlberg’s theory of moral development* (Fig. 2), in which students discussed how the absence of peers or authority figures during exams may cause more cheating. Only 14.7% of students’ open-ended responses (23.9%) included themes that were mapped to *neutralization theory* (Fig. 2), in which students discussed extenuating circumstances that may cause more cheating.

### Paired theories

Overall, we found that students’ responses related to one (*n* = 108) or two (*n* = 71) theories at most, with a few students incorporating themes mapped to three (*n* = 15) or four (*n* = 2) theories. When students’ responses were coded for two theories, the majority of paired theories were between planned behavior theory and game theory (50.7%), and planned behavior theory and Kohlberg’s theory of moral development (35.2%, Table 3). For three theories, the overlap between theories had similar frequencies for each combination (Table 3).

**Table 3:** Percent of paired academic dishonesty theories in students open-ended responses explaining why they believe cheating occurs more frequently online

Paired Theories	Percent
**Two Theories (*n*** **= 71)**	
Planned Behavior and Game Theory	50.7
Planned Behavior and Kohlberg’s Theory of Moral Development	35.2
Planned Behavior and Neutralization Theory	11.3
Kohlberg’s Theory of Moral Development and Neutralization Theory	1.4
Game Theory and Neutralization Theory	1.4
**Three Theories (*n*** **= 15)**	
Planned Behavior, Kohlberg’s Theory of Moral Development and Neutralization Theory	40.0
Planned Behavior, Kohlberg’s Theory of Moral Development and Game Theory	33.3
Planned Behavior, Neutralization Theory and Game Theory	26.7

## Discussion

We found that undergraduate science students believed that willingness to cheat and pressure to cheat were higher online than in-person during the first COVID-interrupted semester. It is concerning that many students’ perceptions of cheating were more pessimistic for online assessments during the COVID-19 pandemic. Cheating online and how learning is assessed during an emergency are pressing issues that must be addressed by institutions as education is likely to become increasingly interrupted by emergencies (Brown et al. 2020). Given the traditionally competitive nature of science courses and the deleterious impact that science exams can have in face-to-face science classes (Eddy et al. 2014; Harris et al. 2019), we must identify interventions to mitigate additional pressure that students might feel during emergency remote learning events. Our data confirmed that the students we surveyed believed cheating occurred more frequently online, which aligned with other research conducted in the early months of the COVID-19 pandemic (e.g., Eaton 2020; Daniels et al. 2021; Lancaster and Cotarlan 2021).

A primary way to reduce the pressure to cheat is to reduce a student’s beliefs that “everyone else is doing it” (Richardson and North 2013). From the 197 students who believed cheating occurred more frequently online and explained their belief, we identified 14 themes that mapped to four academic dishonesty theories. The most frequently discussed themes focused on the differences between the physical space of the online exam “room” and the in-person exam room. Students thought that there were more opportunities for others to cheat online without getting caught, and pointed to the lack of a proctor or professor, along with access to the internet and notes, as the factors that shaped their belief that cheating occurred more online (Supplementary Table C).

### Academic dishonesty theories

We asked science students why and how they believed cheating occurred more frequently online and mapped their responses to four different academic dishonesty theories. This method places the students adjacent to researchers who pick and choose theoretical frameworks to evaluate cheating behavior. In addition to aligning student beliefs to theoretical literature, this also provides insight to develop interventions to help reshape student conceptions of academic cheating, misconduct, and dishonesty. The student open-ended responses that were mapped to multiple academic dishonesty theories (Table 3) show the breadth of explanations that the students had when explaining their beliefs that their peers were cheating more online during an emergency.

Planned behavior theory was by far the most common theory used by students to explain why they thought cheating happened more frequently online. Interestingly, students were five times more likely to discuss the “opportunity to cheat” than the “intention to cheat” aspect of this theory. Lack of a proctor, followed closely by access to the internet, was the most common explanation for an opportunity to cheat. Additionally, students frequently referred to easy access to resources including notes and textbooks. Therefore, up to 86% of science students we surveyed could have their concerns about peers cheating online reduced if their instructors simply moved away from using traditional, closed-book exams. This is consistent with studies from other student populations outside of science (Daniels et al. 2021; Reedy et al. 2021), and our high percentage of students who discussed the potential opportunities to cheat online indicate that moving away from closed-book exams will be an especially impactful intervention in STEM courses.

Game theory was the second most common theory to arise from student responses. While some of these responses discussed strategies that professors used to reduce cheating, such as moving away from closed-book exams, most of these responses focused on students gaming the system or professors failing to take steps to reduce cheating. With 28% of students envisioning cheating as a struggle between surreptitious students and their professors, this suggests that these students are placing the blame on both the professor and the student for cheating. To transform this perspective and quell student concerns about imagined surreptitious behavior, we recommend an emphasis on classroom culture in which professors directly outline their expectations for academic conduct, why they have these expectations, and remain transparent about the decisions they make regarding learning assessments throughout the term.

The third most common theory used by students was Kohlberg’s theory of moral development, with most of these students focused on cheating prevention due to the presence of an authority figure, again placing much of the responsibility on the instructor to control cheating. However, some students recognized the added pressure to cheat online if students believed their peers were cheating. This is further supported by our quantitative results, in which more than 30% of students perceived the pressure to cheat online to be higher than in-person (Table 1, Supplementary Table D). Such sentiments underline the importance of each student’s perception of their peers cheating, as a survey administered to students in face-to-face classes found them to be most influenced to cheat by their peers (Fontaine et al. 2020).

Despite being surveyed only a few months into a global pandemic, only 15% of science students’ responses mapped to neutralization theory. The majority of students who touched on neutralization theory to moralize putative cheating behavior paired this defense with either a theme from planned behavior or game theory - they explained that tumultuous times or lack of educational support may have driven their peers and provided the “how” based on opportunity or surreptitious behavior (Table 3). While it may be easier for students to explain their imagined “how” students cheat more online compared to “why”, the stark contrast between the number of responses that mapped to neutralization theory and the number of responses that mapped to planned behavior theory strongly suggests that most students believed cheating was occurring more online because of the new modality of online learning, rather than the emergency-induced disruption to education. Therefore, *our results indicate that interventions that work in traditional online courses to reduce students concerns of their peers cheating will also be effective in emergency remote courses*.

### Interventions to address student beliefs about peers cheating

#### Address issues of proctoring, internet access, and peer contact

While the high number of students pointing to no proctor as a potential means to cheat online may suggest proctoring software should be utilized, it is expensive and current algorithms discriminate against non-traditional students, students with disabilities, and black and brown students (Swauger 2020). Many of the student responses mapped to game theory cast doubt on the utility of proctoring software, as they describe students finding workarounds. Alternatives that still reduce the perceived problems of proctoring and internet access include higher order exam questions in an open-book format, group oral exams, and final projects. In fact, students who discussed a professor’s steps to prevent cheating typically cited open-book exams.

#### Address academic conduct and reduce pressure to cheat

During and outside of emergencies in education, students should be gently reminded that academic honesty is a critical part of their educational endeavors and that achieving the academic standards of a course without cheating is necessary for the benefit of all. Thus, it is crucial for individual professors, as well as departments and universities, to focus on best practices for educating students on ethical behaviors and how to ensure they are followed. By directly addressing the importance of academic integrity with students, this may mitigate students’ concern that “everyone else is cheating” and quell the pressure to circumvent the system and cheat. An additional step that can be taken to ameliorate student pressure to cheat would be to lower the weight of any given assignment. As one student stated, “The classes that have the most cheating are the ones that hold standards for grades and amount of assignments higher than their students’ well-being.”

#### Examples of successful assessments in the time of COVID-19

With most universities grappling with large-scale online education for the first time due to COVID-19, there are a growing number of examples of instructors rising to the occasion and adopting innovative ways of promoting academic integrity in their virtual classrooms. When instructors at the University of Toronto switched their in-person, short-response exam questions to online, multiple-choice questions, they demonstrated empathy with their students by providing them with practice exams to become acquainted with the new format (Dicks et al. 2020). Some instructors focused on innovative and effective assessment methods suitable for remote instruction methods to discourage cheating and maintain academic integrity (e.g., Raje and Stitzel 2020). Open book tests and the use of supplemental instruction resources became popular, as they encouraged students to think and apply as opposed to recall and reproduce (Cheung Ng 2020).

## Conclusion

We set out to determine if science undergraduate students’ beliefs around peer cheating shifted for online learning during the first COVID-interrupted semester and map their explanations to four academic dishonesty theories. Science students perceived that both willingness to cheat and pressure to cheat were higher in online courses, and the majority of students believed that cheating occurred more frequently online. In explaining their reasoning, the majority of students touched on themes of planned behavior theory—it was so much easier for their peers to cheat online because the modality shifted but means of learning assessment did not. Our results demonstrate that it was a shift in modality, more than the added stresses of the pandemic, that convinced students that cheating was occurring more frequently online. Higher education must reflect inward and address why our students’ perception of academic integrity was worse for online assessments. The prolonged disruption to education brought on by COVID-19 highlighted that instructors and administrators need to reflect on student assessments – their purpose, the message they send to students, and whether students understand why their learning is assessed. Perhaps student perceptions of cheating online are less an indictment on their peers and more an indictment on their class, department, or university for failing to use online assessments for pedagogy and improve classroom culture to foster academic honesty and transparency.

## Supplementary Information

**Additional file 1: **Supplementary Table A. Total number of students attending each institution type. Supplementary Table B. Total number of students for each race/ethnicity category. Supplementary Table C. Total number (*n* = 197) and percent of students that have responses in each subcategory for the four cheating theories from the open-ended responses. Supplementary Table D. The number of students whose Likert scale responses ranked cheating constructs to occur more frequently in person, the same, or more frequently online.

## Data Availability

Data not available due to confidentiality of student information.

## Abbreviation

COVID-19: Coronavirus Disease 2019

## References

[CR1] Ajzen I (1991). The theory of planned behavior. Organ Behav Hum Decis Process.

[CR2] Ajzen I (2002). Perceived behavioral control, self-efficacy, locus of control, and the theory of planned behavior. J Appl Soc Psychol.

[CR3] Arnholt AT, Evans B (2017) BSDA: basic statistics and data analysis. R package version 1.2.0. https://CRAN.R-project.org/package=BSDA

[CR4] Barton DC (2020). Impacts of the COVID-19 pandemic on field instruction and remote teaching alternatives: results from a survey of instructors. Eco Evol.

[CR5] Brown M, McCormack M, Reeves J, Brooks DC, Grajek S (2020) 2020 EDUCAUSE horizon report, teaching and learning edition. EDUCAUSE, p 2020

[CR6] Carpenter D, Harding T, Finelli C, Mayhew M (2005). Work in progress - an investigation into the effect of an institutional honor code policy on academic behavior. Paper presented at proceedings of the 35th annual Frontiers in education conference, Indianapolis, Indiana, 19 October 2005.

[CR7] Castelli FR, Sarvary MA (2021). Why students do not turn on their video cameras during online classes and an equitable and inclusive plan to encourage them to do so. Eco Evol.

[CR8] Cheung Ng CK (2020). Evaluation of academic integrity of online open book assessments implemented in an undergraduate medical radiation science course during COVID-19 pandemic. J Med Imaging Radiat Sci.

[CR9] Crawford J, Butler-Henderson K, Rudolph J, Malkawi B, Glowatz M, Burton R, Magni PA, Lam S (2020) COVID-19: 20 countries’ higher education intra-period digital pedagogy responses. J Appl Learn Teach 3(1):9–28. 10.37074/jalt.2020.3.1.7

[CR10] Daniels LM, Goegan LD, Parker PC (2021). The impact of COVID-19 triggered changes to instruction and assessment on university students’ self-reported motivation, engagement and perceptions. Soc Psychol Educ.

[CR11] Dicks AP, Morra B, Quinlan KB (2020). Lessons learned from the COVID-19 crisis: adjusting assessment approaches within introductory organic courses. J Chem Educ.

[CR12] Dietrich N, Kentheswaran K, Ahmadi A, Teychené J, Bessière Y, Alfenore S, Laborie S, Bastoul D, Loubière K, Guigui C, Sperandio M, Barna L, Paul E, Cabassud C, Liné A, Hébrard G (2020). Attempts, successes, and failures of distance learning in the time of COVID-19. J Chem Educ.

[CR13] Dingwall S (2020). Lessons learned from active engagement in a large-enrollment introductory biochemistry course during a remote quarter. J Chem Educ.

[CR14] DiPietro M (2010). Theoretical frameworks for academic dishonesty. To Improve Acad.

[CR15] Eaton SE (2020). Academic integrity during COVID-19: reflections from the University of Calgary. Int Stud Educ Adm.

[CR16] Eddy SL, Brownell SE, Wenderoth MP (2014). Gender gaps in achievement and participation in multiple introductory biology classrooms. CBE—LSE.

[CR17] England BJ, Brigati JR, Schussler EE (2017). Student anxiety in introductory biology classrooms: perceptions about active learning and persistence in the major. PLoS One.

[CR18] Finn KV, Frone MR (2004). Academic performance and cheating: moderating role of school identification and self-efficacy. J Educ Res.

[CR19] Fontaine S, Frenette E, Hébert M-H (2020). Exam cheating among Quebec’s preservice teachers: the influencing factors. Int J Educ Integr.

[CR20] Goodman AL (2020). Can group oral exams and team assignments help create a supportive student community in a biochemistry course for nonmajors?. J Chem Educ.

[CR21] Harris RB, Grunspan DZ, Pelch MA, Fernandes G, Ramirez G, Freeman S (2019). Can test anxiety interventions alleviate a gender gap in an undergraduate STEM course?. CBE—LSE.

[CR22] Holme TA (2020). Introduction to the journal of chemical education special issue on insights gained while teaching chemistry in the time of COVID-19. J Chem Educ.

[CR23] Holton AJ (2020). Implementation of an emergency multisection online general chemistry curriculum in response to COVID-19 pandemic. J Chem Educ.

[CR24] Huckins JF, daSilva AW, Wang W, Hedlund E, Rogers C, Nepal SK, Wu J, Obuchi M, Murphy EI, Meyer ML, Wagner DD, Holtzheimer PE, Campbell AT (2020). Mental health and behavior of college students during the early phases of the COVID-19 pandemic: longitudinal smartphone and ecological momentary assessment study. J Med Internet Res.

[CR25] Lancaster T, Cotarlan C (2021). Contract cheating by STEM students through a file sharing website: a Covid-19 pandemic perspective. Int J Educ Integr.

[CR26] Levine C, Kohlberg L, Hewer A (1985). The current formulation of Kohlberg’s theory and a response to critics. Hum Dev.

[CR27] Madara DS, Namango SS, Katana H (2016) Theories and models relevant to cheating-behavior. Res Humanit. Sociol Sci 6(17) ISSN 2224–0484

[CR28] Mazer JP, McKenna-Buchanan TP, Quinlan MM, Titsworth S (2014). The dark side of emotion in the classroom: emotional processes as mediators of teacher communication behaviors and student negative emotions. Commun Educ.

[CR29] Nguyen JG, Keuseman KJ, Humston JJ (2020). Minimize online cheating for online assessments during COVID-19 pandemic. J Chem Educ.

[CR30] Pajares MF (1992). Teacher’s beliefs and educational research: cleaning up a messy construct. Rev Educ Res.

[CR31] Perets EA, Chabeda D, Gong AZ, Huang X, Fung TS, Ng KY, Bathgate M, Yan ECY (2020). Impact of the emergency transition to remote teaching on student engagement in a non-STEM undergraduate chemistry course in the time of COVID-19. J Chem Educ.

[CR32] Petillion RJ, McNeil WS (2020). Student experiences of emergency remote teaching: impacts of instructor practice on student learning, engagement, and well-being. J Chem Educ.

[CR33] Putarek V, Pavlin-Bernardic N (2020). The role of self-efficacy for self-regulated learning, achievement goals, and engagement in academic cheating. Euro J Psych Educ.

[CR34] Raje S, Stitzel S (2020) Strategies for effective assessments while ensuring academic integrity in general chemistry courses during COVID-19. J Chem Educ 97(9):3436–3440. 10.1021/acs.jchemed.0c00797

[CR35] Reedy A, Pfitzner D, Rook L, Ellis L (2021). Responding to the COVID-19 emergency: student and academic staff perceptions of academic integrity in the transition to online exams at three Australian universities. Int J Educ Integr.

[CR36] Richardson R, North M (2013). Strengthening the trust in online courses: a common sense approach. J Comput Sci Coll.

[CR37] Roberson PK, Shema SJ, Mundfrom DJ, Holmes TM (1995). Analysis of paired Likert data: how to evaluate change and preference questions. Fam Med.

[CR38] Rupnow RL, LaDue ND, James NM, Bergan-Roller HE (2020). A perturbed system: how tenured faculty responded to the COVID-19 shift to remote instruction. J Chem Educ.

[CR39] Spaulding M (2009). Perceptions of academic honesty in online vs. face-to-face classrooms. J Interact Online Learn.

[CR40] Storch J, Storch E, Clark P (2002). Academic dishonesty and neutralization theory: a comparison of intercollegiate athletes and nonathletes. J Coll Stud Dev.

[CR41] Supiano B (2020) Teaching: why (some) professors are so worried about cheating. Chronicle of Higher Education (29 Oct 2020): https://www.chronicle.com/newsletter/teaching/2020-10-29

[CR42] Swauger S (2020) Our bodies encoded: algorithmic test proctoring in higher education. In: Stommel J, Friend C, Morris SM (eds) Critical digital pedagogy. Hybrid Pedagogy Inc.

[CR43] Sykes GM, Matza D (1957). Techniques of neutralization: a theory of delinquency. Am Sociol Rev.

[CR44] Turner S, Uludag S (2013) Student perceptions of cheating in online and traditional classes. Paper presented in Frontiers in education conference, Oklahoma City, Oklahoma, 23-26 October 2013. 10.1109/FIE.2013.6685007

[CR45] van Zyl A, Thomas A (2015). Academic honesty: perceptions of millennial university students and the role of moderating variables. KOERS - Bull Christ Scholarsh.

[CR46] Walsh LL, Arango-Caro S, Wester ER, Callis-Duehl K (2021). Training faculty as an institutional response to COVID-19 emergency remote teaching supported by data. CBE—LSE.

[CR47] Wester ER, Walsh LL, Arango-Caro S, Callis-Duehl K (2021) Student engagement declines in STEM undergraduates during COVID-19 driven remote learning. J Microbiol biol Educ 22(1). 10.1128/jmbe.v22i1.2385 10.1128/jmbe.v22i1.2385 PMC8046661 33884093

