# Supplementary material for: Why and how science students in the United States think their peers cheat more frequently online: perspectives during the COVID-19 pandemic
Source: Int J Educ Integr. 2021 Nov 17;17(1):23. doi: 10.1007/s40979-021-00089-3 (PMC8594954; doi:10.1007/s40979-021-00089-3)
Supplement: Supplementary file 1 — Additional file 1: Supplementary Table A. Total number of students attending each institution type. Supplementary Table B. Total number of students for each race/ethnicity category. Supplementary Table C. Total number (n = 197) and percent of students that have responses in each subcategory for the four cheating theories from the open-ended responses. Supplementary Table D. The number of students whose Likert scale responses ranked cheating constructs to occur more frequently in person, the same, or more frequently online. [file 40979_2021_89_MOESM1_ESM.docx]

**Why and how science students in the United States think their peers cheat more frequently online: perspectives during the COVID-19 pandemic**

**Supporting Information**

Supplementary Table A

Supplementary Table B

Supplementary Table C

Supplementary Table D

**Supplementary Table A**. Total number of students attending each institution type.

| **Institution Type** | **Total Number** |
| --- | --- |
| Community College | 18 |
| Master’s Granting Institution, non-research intensive | 25 |
| PhD Granting Institution, non-research intensive | 22 |
| Primarily Undergraduate-serving Institution | 57 |
| R1 Research Intensive Institution | 146 |
| R2 Research Intensive Institution | 28 |

**Supplementary Table B**. Total number of students for each race/ethnicity category.

| **Race/Ethnicity** | **Total Number** |
| --- | --- |
| American Indian, Alaska Native, Native Hawaiian or Other Pacific Islander | 1 |
| Asian American, Asian, or Filipinx | 78 |
| Asian American, Asian, or Filipinx, Middle Eastern or North African | 1 |
| Black or African American | 7 |
| Black or African American, Asian American, Asian, or Filipinx | 1 |
| Hispanic or Latinx | 60 |
| Hispanic or Latinx, Asian American, Asian, or Filipinx | 2 |
| Middle Eastern or North African | 9 |
| Multi-ethnic Hispanic and Caucasian | 1 |
| White or Caucasian | 111 |
| White or Caucasian, American Indian, Alaska Native, Native Hawaiian or Other Pacific Islander | 3 |
| White or Caucasian, Asian American, Asian, or Filipinx | 4 |
| White or Caucasian, Asian American, Asian, or Filipinx, Middle Eastern or North African | 1 |
| White or Caucasian, Black or African American | 5 |
| White or Caucasian, Hispanic or Latinx | 10 |
| White or Caucasian, Middle Eastern or North African | 1 |
| Prefer not to answer | 4 |

**Supplementary Table C**. Total number (*n* = 197) and percent of students that have responses in each subcategory for the four cheating theories from the open-ended responses.

| **Description of the Subcategory** | **Total Number** | **Percent** | **Example** |
| --- | --- | --- | --- |
| **Kohlberg’s Theory of Moral Development** | | | |
| ­ | 35 | 17.8 | “When taking online tests or completing projects you can have the notes beside of you [sic]. In class this is not possible when the teacher is watching you take the tests, or complete the assignments.” |
| Conventional: Pressure to Cheat | 14 | 7.1 | “There is more motivation or pressure to cheat due to lack of motivation for class work, but still desiring a good grade.” |
| Conventional: Exam room with peers | 9 | 4.6 | “You are surrounded by more people in class.” |
| **Planned Behavior** | | | |
| Actions Indicating Plan to Cheat | 30 | 15.2 | “I think there greater [sic] temptation to cheat at home because there isn’t the pressure that professors add by being there.” |
| Opportunities to Cheat without getting caught | 169 | 85.8 | See quotes in the five rows below for specific opportunity examples |
| Easy | 31 | 15.7 | “It's easier to cheat, and people have pressures that make it harder to concentrate on work.” |
| Resources and notes | 57 | 28.9 | “More likely to check the book to verify answers on quizzes or exams” |
| Internet | 61 | 31.0 | “The internet is at our finger tips and the temptation is high” |
| Communication with Classmates | 18 | 9.1 | “Some classes do not require a webcam, where students work on the test together when it's not allowed” |
| No proctor | 77 | 39.1 | “There is no way to monitor what is in the students' environment throughout the entire test” |
| **Neutralization Theory** | | | |
| Denial of responsibility/victim or Coping Strategy | 29 | 14.7 | “In classes where the same or more amount of work is expected (because we "have more time now") is when students cheat to get by. Students are becoming overwhelmed, losing motivation to keep up, and even despairing when there are several other stressors going on in their lives. Feeling the need for good grades in order to be good enough has always been a pressure to cheat. But now there is no motivation to keep up or pay attention because we are stuck at home waiting for the next day to be the same.” |
| **Game Theory** | | | |
| Students being tricky or sly when cheating | 31 | 15.7 | “I know some people who smear their webcam to decrease the quality of proctored exams so their eyes can move around to cheat more easily; hiding cheat sheets not visible by webcam; collaborating with others” |
| Professors changing for online | 19 | 9.6 | “It's best when professors have open-book open-note exams because then everyone starts on a more level playing field.” |
| Professors missteps | 20 | 10.2 | “A lot of professors just don’t use the 3^rd^ party apps for test/quiz monitoring and students don’t feel like classes are worth as much effort” |

**Supplementary Table D**. The number of students whose Likert scale responses ranked cheating constructs to occur more frequently in person, the same, or more frequently online.

| **Cheating construct** | **Higher in-person** | **Same** | **Higher online** |
| --- | --- | --- | --- |
| Willingness to cheat | 6 | 188 | 104 |
| Pressure to cheat | 14 | 192 | 90 |
